# A randomized, fellow eye, comparison of keratometry, aberrometry, tear film, axial length and the anterior chamber depth after eye rubbing in non-keratoconic eyes

**DOI:** 10.1186/s40662-017-0084-8

**Published:** 2017-08-14

**Authors:** Jordan V. Chervenkoff, Elizabeth Hawkes, Gabriela Ortiz, Deborah Horney, Mayank A. Nanavaty

**Affiliations:** Sussex Eye Hospital, Brighton & Sussex University Hospitals NHS Trust, Eastern Road, Brighton, BN2 5BF UK

**Keywords:** Eye rubbing, Ectasia, Tear film break up time, Astigmatism, Aberrations

## Abstract

**Background:**

To investigate the effect of eye rubbing on keratometry (K), aberrometry, tear film break-up-time (TFBUT) and anterior chamber depth (ACD).

**Methods:**

Volunteers without any corneal pathology or dry eyes were randomised to rubbing in one eye and the fellow-eye was control. Eye rubbing was performed for 2 min. Primary outcomes studied were anterior and posterior K changes. Secondary outcomes were changes in TFBUT, axial length (AL) & ACD, K changes in various zones, asphericity and aberrometry. Pre and post rubbing K, aberrometry, ACD and TFBUT were assessed in a predetermined sequence. The relationship of the above parameters to axial length (AL) was also assessed. Astigmatism was analysed using vector analysis.

**Results:**

Pre versus post rubbing, anterior flatter K further flattened (42.51 ± 1.52 D vs. 42.36 ± 1.53 D, *p* = 0.003) and the changes to J0 vector in central cornea (−0.16 ± 0.26 D vs. -0.27 ± 0.33 D, *p* = 0.038) suggested change to against-the-rule (ATR) astigmatism. There was significant change in Z_2_^+2^ polynomial following rubbing. We found a positive correlation between axial length and change in posterior K (*r* = 0.335, *p* = 0.020). The TFBUT reduced following eye rubbing (15.3 s vs. 13.9 s, *p* = 0.0001). There was a positive correlation between AL and increase in ACD post rubbing (*r* = 0.300, *p* = 0.038). There was a positive correlation between ACD and change in mean posterior K (*r* = 0.305, *p* = 0.035).

**Conclusions:**

In healthy eyes, following eye rubbing, there is a significant change in TFBUT and central anterior K changes towards ATR astigmatism. Longer eyes had more changes in posterior K and ACD. Whereas, eyes with deeper ACD showed more steepness of posterior K.

**Trial registration:**

ClinicalTrials.gov ID: NCT02131740.

**Electronic supplementary material:**

The online version of this article (doi:10.1186/s40662-017-0084-8) contains supplementary material, which is available to authorized users.

## Background

Several biochemical and biomechanical processes link eye rubbing to structural corneal changes and ectatic corneal disorders like keratoconus, keratoglobus and pellucid marginal degeneration, [1, 2] especially in patients suffering from dry eyes or atopy [3–5]. Eye rubbing has been reported to alter the surface regularity prior to topography and this was thought to be due to changes in tear film and/or corneal remodelling [6]. However, the detailed impact of eye rubbing on the anterior segment of a healthy eye is uncertain. Understanding how the healthy eye’s anterior segment property changes following eye rubbing may provide some insight into the understanding the impact of eye rubbing on abnormal anterior segment measurements and its impact on aetiology of corneal and anterior segment disorders (like keratoconus) in non-healthy eyes.

We conducted a randomised, fellow eye controlled study, to investigate the changes in keratometry (K), aberrometry, tear film break-up-time (TFBUT) and anterior chamber depth (ACD) following eye rubbing in healthy volunteers. We also aimed to analyse the relationship between the above parameters with age and axial length (AL) of the eyeball.

## Methods

This prospective single-centre, randomised, fellow-eye controlled study (http://clinicaltrials.gov/show/NCT02131740) was conducted at the Sussex Eye Hospital, Brighton & Sussex University Hospitals NHS Trust in Brighton, United Kingdom in March and April 2014. This study was approved by the National Ethics Committee and followed the tenets of the Declaration of Helsinki. Internal email advertising the recruitment for this study was sent to 3000 employees of the Brighton and Sussex University Hospitals NHS trust. The volunteers were requested to contact via email or internal phone to arrange an appointment. Prior to the study appointment, all volunteers were sent an information sheet detailing the study.

The participants were consented for taking part in this study by signing a form. Volunteers with known history of corneal ectasia (like keratoconus, etc.) and pre-existing ocular abnormalities or diagnoses, concurrent use of any topical drops including lubricating eye drops, contact lens wearers, previous surgery or history of atopy or eczema were excluded. Any included volunteer who was noted to have any signs of forme-fruste keratoconus (steep posterior elevation and/or irregular bow tie pattern on axial map) on topography were not subjected to any further tests and were excluded.

An online random number generator was used to randomise the eyes of the subjects to digital rubbing in one eye (either left or right eye). Eye rubbing was performed by the same researcher (EDH) for 1 min followed by 5 s break and a further 1 min using the index finger of the right hand in a circular, clockwise motion over a closed eyelid. Volunteers were instructed to look straight ahead to maintain the primary position of gaze with the fellow eye, which was a control. The fellow eye (control) was undisturbed. A video demonstrating the rubbing methodology can be found online (Additional file 1: Video S1). A second masked observer performed all other measurements.

The subjects underwent the examinations in the following sequence for each eye:

1. Partial coherence interferometry for axial length (AL) and optical anterior chamber depth (ACD) measurement on the IOLMaster® (Zeiss Meditec, Germany).
2. Scheimpflug scan of the eye on the Scheimpflug tomographer (Pentacam®, Oculus, Germany).
3. The TFBUT assessment through the slit lamp located next to the Scheimpflug tomographer.
4. Whilst they were on the seat for assessment on the Scheimpflug tomographer, the eye was rubbed as per the protocol described above by the masked observer.
5. A repeat Scheimpflug scan was performed immediately on each eye without moving the patient from the seat.
6. This was immediately followed by TFBUT assessment which was performed on the slit lamp.
7. This was followed by a repeat measurement of AL and ACD on the IOLMaster®.

To minimise the duration between rubbing and scanning, the intervention was performed on the chair of the Scheimpflug tomographer (Pentacam, Oculus, Germany). Observers were masked and made conscious efforts to hide the identity of the rubbed and non-rubbed eye from each other. Topical anaesthetic drop was not used prior to eye rubbing as it may affect the TFBUT. Moreover, as the TFBUT was assessed, intraocular pressure measurement was not included as a study outcome measure. Only one eye was randomised and rubbed but the measurements were performed for both eyes (other eye as control).

Data on the following outcome measures were collected pre and post rubbing in experimental and control eyes (in this sequence):

### Primary outcome

Keratometric changes in anterior and posterior cornea pre and post rubbing as compared to a fellow eye as control.

### Secondary outcomes

1. *Tear film break-up time (TFBUT):* Tear film break up time was assessed using one drop of 2% Fluorescein (Fluorescein minims, Bausch and Lomb, USA) in each eye using a cobalt blue filter on the slit lamp biomicroscope.
2. *Axial length (AL) and anterior chamber depth (ACD)* were measured by partial coherence interferometry (IOLMaster®, Carl Zeiss, Germany). Correlation of AL and ACD with age, changes in anterior and posterior keratometry. AL was measured from the anterior corneal surface.
3. *Scheimpflug Holladay equivalent keratometry (EKR) in the central (3 mm), paracentral (4-5 mm) and peripheral (6-7 mm) zones*. The detailed Holladay Equivalent K report of the Pentacam machine gives equivalent keratometry at various optical scan diameters. For the purpose of this study, the keratometric values for the central (3 mm), paracentral (4-5 mm) and peripheral (6-7 mm) zones were collected.
4. *Anterior asphericity (Qant), posterior asphericity (Qpost), pachymetry apex, minimum pachymetry and corneal volume*.
5. *Changes in corneal Zernike wave-front aberrations (WFA):* Total high-order aberrations and the total low-order aberrations for the anterior, posterior and total cornea was collected on the Pentacam. The mean-root square (RMS) error up to the 4th order was recorded.

### Data analysis

All data was entered onto an Excel Spread sheet (Microsoft Office 2011, USA) and analysed using StatPlus: Mac Pro 2016 (AnalystSoft Inc., USA). Normality of the data was tested using Kolmogorov-Smirnov test. Data was analysed using paired t-tests of means comparing quantitative outcomes in experimental (rubbed) and control eyes, pre and post rubbing. Astigmatism was analysed using power vectors (J0 and J45) [7]. The method is described elsewhere [7]. In this vectoral analysis, described by Thibos and Horner, [7] any refractive or keratometric error can be expressed as a combination of 3 orthogonal components; that is, M, J0, and J45. In common clinical terms, M is the spherical equivalent (SE) and is not relevant for the purposes of studying astigmatism. The J0 component expresses the power of a Jackson cross-cylinder with its axes at 180 degrees and 90 degrees. The J45 component expresses the power of a Jackson cross-cylinder with its axes at 45 degrees and 135 degrees (oblique astigmatism). Pearson’s correlation was calculated where a correlation was assessed. *P* value of less than 0.05 was considered statistically significant.

## Results

The study involved 98 eyes (49 patients). The mean age was 43.3 ± 12.3 years. There were 47 Caucasians, 1 Asian and 1 Afro-Caribbean. There were 27 (55%) female and 22 (45%) males. No one was excluded. No immediate complication or unintended effects were observed in any eye during the study. No patients were referred to acute ophthalmology services due to the discovery of any new pathology (e.g., forme-fruste keratoconus, etc.).

### Primary outcome

- *Anterior keratometry*

Pre versus post eye rubbing, the flattest anterior keratometry became significantly flatter in case eyes after rubbing (*p* = 0.003) unlike the control eyes (*p* = 0.087). However, there was no significant change in the steepest anterior keratometry in experimental (*p* = 0.558) and control (*p* = 0.459) eyes. There was no significant change in the maximum anterior K (Kmax) in experimental (*p* = 0.223) and control eyes (*p* = 0.116; Table 1). There was a significant change in J0 (*p* = 0.003) post rubbing in experimental eyes only (Table 1).

(b) Posterior keratometry

**Table 1:** Parameters pre vs. post rubbing in case and control eyes

		Cases		*p*-value*	Controls		*p*-value*
		Pre-rubbing	Post-rubbing		Pre-rubbing	Post-rubbing	
Anterior Keratometry (D) (Mean ± SD)	Mean K1	42.51 ± 1.52 (38.80; 46.30)	42.36 ± 1.53 (38.50; 45.40)	0.003	42.51 ± 1.49 (39.00; 45.70)	42.60 ± 1.48 (38.90; 45.80)	0.087
	Mean K2	43.36 ± 1.73 (39.70; 47.80)	43.40 ± 1.61 (39.70; 46.80)	0.558	43.42 ± 1.64 (39.60; 46.80)	43.47 ± 1.54 (39.80; 46.00)	0.459
	Mean Kmax	44.19 ± 1.75 (40.30; 47.70)	43.46 ± 1.92 (40.40; 50.90)	0.223	44.18 ± 1.61 (40.00; 47.40))	44.36 ± 1.68 (40.70; 49.40)	0.116
Posterior Keratometry (D) (Mean ± SD)	Mean K1	−6.07 ± 0.24 (−6.70; −5.60)	−6.07 ± 0.25 (−6.70; −5.60)	0.799	−6.09 ± 0.25 (−6.80; −5.60)	−6.08 ± 0.24 (−6.80; −5.50)	0.699
	Mean K2	−6.24 ± 0.90 (−7.10; −0.40)	−6.36 ± 0.28 (−7.00; −0.40)	0.324	−6.38 ± 0.29 (−7.20; −5.70)	−6.11 ± 1.82 (−7.20; 6.20)	0.281
	Mean Kmax	44.19 ± 1.75 (40.40; 47.70)	44.36 ± 1.92 (40.40; 50.90)	0.223	44.18 ± 1.61 (40.00; 47.40)	44.36 ± 1.68 (40.70; 49.40)	0.116
Overall anterior corneal power vectors (D)	J0	−0.33 ± 0.32	−0.43 ± 0.36	0.004	−0.38 ± 0.29	−0.37 ± 0.28	0.477
	J45	−0.02 ± 0.19	−0.03 ± 0.23	0.334	0.02 ± 0.20	0.01 ± 0.19	0.374
Overall posterior corneal power vectors (D)	J0	−0.13 ± 0.06	−0.14 ± 0.06	0.333	−0.14 ± 0.06	−0.13 ± 0.07	0.521
	J45	−0.00 ± 0.05	−0.00 ± 0.05	0.654	0.01 ± 0.06	0.01 ± 0.06	0.939
ACD (mm) (Mean ± SD)		2.85 ± 0.39 (2.12; 3.77)	2.85 ± 0.39 (2.07; 3.74)	0.057	2.85 ± 0.39 (2.10; 3.83)	2.85 ± 0.39 (3.84; 2.10)	0.601
AL (mm) (Mean ± SD)		23.71 ± 1.05 (21.26; 26.42)	23.73 ± 1.11 (21.54; 27.30)	0.124	23.71 ± 1.06 (21.26; 26.42)	23.78 ± 1.09 (21.54; 27.30)	0.133
TBUT (sec) (Mean ± SD)		5.31 ± 2.58 (2.00; 10.00)	3.92 ± 2.32 (1.00; 10.00)	<0.001	5.31 ± 2.66 (2.00; 10.00)	4.86 ± 2.49 (1.00; 10.00)	0.096

*Abbreviations*: *D* = Diopters; *ACD* = Anterior chamber depth; *AL* = Axial Length; *mm* = millimetres; *SD* = standard deviation * paired t test

Pre versus post rubbing, there was no significant difference in the mean flattest posterior keratometry in experimental (*p* = 0.799) and in control eyes (*p* = 0.699). Furthermore, the mean steepest posterior corneal keratometry was not significantly different in case (*p* = 0.324) and in controls (*p* = 0.281) as well. There was no significant change in the maximum posterior K (Kmax) in experimental (*p* = 0.223) and control eyes (*p* = 0.116; Table 1). There was no significant difference in posterior keratometry J0 and J45 (Table 1).

### Secondary outcomes

1. Tear-film break-up time

Pre versus post rubbing, the TFBUT decreased from 15.3 s to 13.9 s in experimental (*p* = 0.0001) eyes only, compared to 15.3 vs. 14.8 s (*p* = 0.096) in controls.

2) Axial length (AL) and anterior chamber depth (ACD)

There was no significant difference in AL between experimental and control eyes (*p* = 0.407). There was positive correlation between AL and age in experimental (*r* = 0.371; *p* = 0.009) and in control eyes (*r* = 0.366; *p* = 0.011). No correlation was found between the AL vs. the changes in mean anterior keratometry in experimental compared to control eyes. However, there was a positive correlation between the AL vs. the change in mean posterior keratometry in experimental eyes only (*r* = 0.335; *p* = 0.020) (i.e., if the AL was increased, the change in mean posterior K was greater).

There was no significant difference in ACD pre and post rubbing in any groups (Table 1). A positive correlation was found in experimental eyes only between the AL and increase in the ACD pre and post eye rubbing (Pearson’s correlation coefficient = 0.3; *p* = 0.038). However, although not statistically significant, the average ACD increased in experimental eyes (Table 1). No correlation was found between the ACD vs. the changes in mean anterior keratometry in experimental and control eyes. However, there was a positive correlation between the ACD vs. the changes in mean posterior keratometry in experimental eyes only (*r* = 0.305; *p* = 0.035) (i.e., if the ACD was increased, the change in mean posterior K was greater).

3) Keratometry in central, paracentral and peripheral cornea

Further analysis of keratometric vectors in the central, paracentral and peripheral cornea (Table 2) revealed significant changes in J0 in the central zones of experimental eyes with a change towards against-the-rule astigmatism (*p* = 0.038) (Table 2).

4) Asphericity, pachymetry and corneal volume

**Table 2:** Power vectors for the central, paracentral and peripheral corneal zones

	CENTRAL (1–3 mm)				PARACENTRAL (4–5 mm)				PERIPHERAL (6–7 mm)			
	CASES		CONTROLS		CASES		CONTROLS		CASES		CONTROLS	
	J0	J45	J0	J45	J0	J45	J0	J45	J0	J45	J0	J45
Mean power (D ± SD) Pre-rubbing	−0.16 ± 0.26 (−1.09; 0.27)	−0.03 ± 0.25 (−1.12; 0.44)	−0.18 ± 0.25 (−0.99; 0.24)	0.02 ± 0.21 (−0.40; 0.58)	−0.31 ± 0.28 (−1.16; 0.20)	−0.01 ± 0.23 (−0.56; 0.68)	−0.29 ± 0.23 (−0.92; 0.10)	0.04 ± 0.19 (−0.28; 0.77)	−0.34 ± 0.34 (−1.29; 0.42)	−0.02 ± 0.21 (−0.69; 0.64)	−0.27 ± 0.35 (−1.22; 0.67)	0.02 ± 0.17 (−0.27; 0.62)
Mean power (D ± SD) Post-rubbing	−0.27 ± 0.33 (−1.62; 0.16)	−0.05 ± 0.29 (−0.93; 0.41)	−0.23 ± 0.26 (−1.00; 0.29)	0.01 ± 0.23 (−0.47; 0.45)	−0.32 ± 0.38 (−1.22; 0.97)	−0.02 ± 0.23 (−0.57; 0.51)	−0.24 ± 0.31 (−0.88; 0.65)	0.00 ± 0.18 (−0.41; 0.47)	−0.37 ± 0.37 (−1.22; 0.47)	−0.02 ± 0.19 (−0.52; 0.30)	−0.27 ± 0.37 (−0.94; 0.75)	0.02 ± 0.18 (−0.41; 0.55)
Mean power Difference (D)	−0.11	−0.02	−0.05	−0.01	−0.01	−0.01	0.05	−0.04	−0.03	0.00	0.00	0.00
*P*-value*	0.038	0.490	0.333	0.916	0.794	0.854	0.266	0.081	0.494	0.905	0.992	0.746

*Abbreviations*: *D* = Diopters; *SD* = standard deviation

*paired t test

Pre and post rubbing, anterior Q-value did not change in the experimental eyes (−0.307 ± 0.13 vs. -0.310 ± 0.12, *p* = 0.822) and controls (−0.344 ± 0.13 vs. -0.318 ± 0.16, *p* = 0.112). This was similar for posterior Q-value in experimental eyes (−0.382 ± 0.15 vs. -0.392 ± 0.15, *p* = 0.182) and controls (−0.391 ± 0.17 vs. -0.396 ± 0.16, *p* = 0.606).

Pre and post rubbing, pachymetry did not change significantly in experimental (575.4 ± 32.9 μm vs. 575.2 ± 31.4 μm, *p* = 0.950) and in control eyes (576.1 ± 30.7 μm vs. 574.8 ± 33.9 μm, *p* = 0.606).

Corneal volume did not change significantly pre and post rubbing in experimental (62.7 ± 3.4 mm^3^ vs. 62.8 ± 3.9 mm^3^, *p* = 0.847) and in control eyes (63.0 ± 3.5 mm^3^ vs. 61.4 ± 9.4 mm^3^, *p* = 0.205).

5) Corneal aberrations

Statistically significant differences before and after rubbing were found in experimental eyes for the Z_2_^+2^ aberration for anterior cornea only. The average anterior corneal Z_2_^+2^ RMS became more negative by 0.159 μm post-rubbing (−0.753 μm pre vs. -0.912 μm post, *p* = 0.001) resulting in an increase in the entire corneal Z_2_^+2^ RMS by 0.16 ± 0.52 μm (*p* = 0.003). There was no statistically significant difference in any other Zernike’s coefficient for the anterior or posterior cornea (Table 3).

**Table 3:** Zernike’s polynomials up to the 4th order in cases and controls

Zernike’s Polynomial	Cases			Controls		
	*P* values comparing pre vs. post rubbing			*P* values comparing pre vs. post rubbing		
	WFA front	WFA back	WFA cornea	WFA front	WFA back	WFA cornea
Z^0^_0_ (Piston)	0.309	0.417	0.220	0.440	0.432	0.220
Z^1^_1_ (Tilt (Prism))	0.164	0.306	0.215	0.605	0.567	0.552
Z^−1^_1_ (Tilt (Prism))	0.257	0.336	0.229	0.339	0.275	0.401
Z^2^_2_ (Astigmatism)	0.001	0.317	0.003	0.678	0.244	0.515
Z^0^_2_ (Defocus)	0.315	0.351	0.282	0.453	0.349	0.560
Z^−2^_2_ (Astigmatism)	0.902	0.824	0.934	0.720	0.692	0.630
Z^3^_3_ (Trefoil)	0.772	0.238	0.973	0.699	0.653	0.586
Z^1^_3_ (Horizontal Coma)	0.070	0.273	0.088	0.282	0.365	0.253
Z^−1^_3_ (Vertical Coma)	0.344	0.166	0.308	0.520	0.641	0.496
Z^−3^_3_ (Trefoil)	0.813	0.909	0.857	0.869	0.566	0.754
Z^4^_4_ (Quadrafoil)	0.157	0.861	0.176	0.342	0.853	0.399
Z^2^_4_ (Secondary Astigmatism)	0.193	0.404	0.226	0.257	0.132	0.357
Z^0^_4_ (Spherical aberration)	0.230	0.071	0.191	0.716	0.845	0.689
Z^−2^_4_ (Secondary Astigmatism)	0.430	0.809	0.467	0.591	0.848	0.565
Z^−4^_4_ (Quadrafoil)	0.828	0.057	0.530	0.774	0.594	0.840

*Abbreviation*: *WFA* = wavefront aberration

## Discussion

While previous research has explored the structural and histological changes on a microscopic level in the cornea following eye rubbing, [8–14] our research focused on the link between keratometric changes, aberrometry, TFBUT and ACD occurring because of digital rubbing in healthy eyes. We found a significant change in the anterior cornea only. Anterior keratometry showed further flattening and changes to the J0 vector, suggesting a trend toward against-the-rule (ATR) astigmatism following eye rubbing. The TFBUT reduced following eye rubbing. We also found a positive correlation between AL and change in posterior keratometry. There was a positive correlation between AL and increase in ACD post rubbing.

A previous study by Mansour & Haddad explored topographic corneal changes immediately and 5 min after eye rubbing for 1 min [10]. They measured a statistically significant increase in the induced astigmatism up to 0.74 D (*p* = 0.04) post 1 min rubbing. A significant increase was noted in the surface regularity and surface asymmetry indices as well. However, astigmatic vectors were not assessed in their study and the duration of assessments were different too. In our study, vector analysis showed negative changes in the J0 component in the anterior and, to a lesser extent, the posterior cornea of rubbed eyes, demonstrating a tendency towards against-the-rule (ATR) astigmatism after rubbing. The increase in the ATR astigmatism was statistically significant in the central zone of the anterior cornea only. This finding is interesting as there is enough evidence now that keratoconic patients manifest ATR myopic astigmatism at presentation, which later becomes irregular astigmatism [15]. We hypothesise this change in astigmatism was caused by structural changes in the central cornea. There is some evidence that eye rubbing is likely to lead to transient keratometric changes in the central zone first where the cornea is thinnest [9, 11]. It is also known that the rubbing induces transient thinning of the central and mid-peripheral epithelium, which returned to baseline thickness in 15 to 45 min (quicker recovery centrally) [12]. The thinner cornea is less resistant to intraocular pressure forces in the anterior chamber, which explains the increase in the ACD we detected (Table 3). Keratometric changes in the peripheral cornea might occur later in the pathological process after prolonged eye rubbing, likely due to the increased thickness peripherally compared to the central zone.

We did not find any significant changes in posterior corneal keratometry. Although posterior elevation is considered a significant differentiating factor between normal and keratoconic eyes, its reliability to detect subclinical ectasia is low [16]. From the findings of our study, we can postulate that it is possible that the changes in the anterior corneal curvature precede the changes in posterior corneal curvature after eye rubbing. Again, this can be multifactorial, as on further assessment of relationship of AL and ACD to the study parameters, we found some correlation between the AL and ACD vs. mean change in posterior keratometry, suggesting that longer eye and eyes with deeper ACD may have more changes in posterior keratometery after eye rubbing. This needs to be researched in future with controlled studies.

Statistically significant changes in corneal wavefront aberrations were observed in the anterior corneal astigmatism (Z_2_^+2^) and the total corneal wavefront aberrations (Table 3). Our results showed that lower order aberrations changed significantly in the anterior cornea. However, there was minimal and non-statistically significant change in lower order aberration in the posterior cornea. The changes observed in the J0 astigmatism vector may have some bearing with the above findings in aberrations. A plausible reason for these changes could be change in the tear film or due to transient corneal epithelial remodelling following rubbing. Like Osuagwa and Alanazi [17], we did not find any difference in pachymetry of the corneal apex after eye rubbing in healthy volunteers, so epithelial remodelling seems to be a less plausible aetiology of our findings. Again, our study did not include specific tests to look solely at the epithelial thickness pre and post rubbing.

Previous research suggested no change in TFBUT following eye rubbing in healthy eyes [18]. However, we found a statistically significant decrease of 1.4 s in the TFBUT parameter following rubbing. This could be due to rubbing-associated irritation of the meibomian glands or distortion of the ducts leading to a disruption of the outermost lipid layer allowing for quicker evaporation of the aqueous component [19]. A similar study on rats by Greiner et al. [20] using digital eye rubbing with pressure for 5 min also suggested that the changes start in the conjunctival epithelium within seconds, which leads to a rapid inflammatory cascade. Due to this reason, we agreed and decided on fixing the duration of eye rubbing to 1 min with a 5-s break followed by an additional minute of eye rubbing.

The strength of this study is that it was a prospective, randomised, fellow eye controlled study of healthy volunteers, the same observer rubbed all the eyes, a masked observer performed all measurements and the rubbing was done in primary straight position of the eyeball under the closed eyelids. Another strength of our study is that it looked at multiple outcomes giving a wider and real picture of the immediate changes. Due to many outcome measures assessed in healthy volunteers in this study, it was not possible to repeat all these measurements at frequent time intervals post rubbing and perhaps this may be the limitation of this study. We did not include IOP as our outcome measure as applanation tonometry may lead to surface irregularity, which may affect TFBUT and Schiempflug scanning. We standardized the rubbing technique in this study and had a single observer rub the eyes, but the severity of the eye rubbing may have varying changes on the study parameters. Moreover, we cannot rule out the theoretical possibility of fluorescein tear film disturbance when it is instilled prior to TFBUT measurements.

## Conclusions

In summary, eye rubbing of healthy eyes induces a significant change in astigmatism and tear film behaviour. There were significant changes in ATR astigmatism in the central cornea following eye rubbing. Axial myopia (increased AL) was associated with more change in posterior K and deeper ACD post eye rubbing. Whereas, eyes with deeper ACD showed more changes in posterior K.

## Additional file

Additional file 1: Video S1. On the method of eye rubbing used in this study. (MP4 1405 kb)
